# The potential of shallot skin powder and actinomycetes metabolites as antimicrobe and antibiofilm in the treatment of eel (*Anguilla bicolor bicolor*) infected with *Aeromonas hydrophila*

**DOI:** 10.1186/s13104-023-06611-9

**Published:** 2023-11-09

**Authors:** Dinamella Wahjuningrum, Aisyah Hilal, Diana Elizabeth Waturangi, Sri Nuryati

**Affiliations:** 1 Department of Aquaculture, IPB University, Dramaga Street, Bogor, 16680 Indonesia; 2 Faculty of Biotechnology, Atma Jaya Catholic University of Indonesia, Jalan Raya Cisauk-Lapan No. 10, Sampora, Cisauk, Tangerang, Banten 15345 Indonesia

**Keywords:** Shallot skin powder, Actinomycetes, *Aeromonas hydrophila*, *Anguilla bicolor bicolor*, Antibiofilm, Antimicrobe

## Abstract

**Background:**

Eel (*Anguilla bicolor bicolor*) is an Indonesian export commodity. However, it is facing a problem related to *Aeromonas hydrophila*, which can cause motile aeromonas septicemia (MAS) and produce biofilm formation. Problem with antibiotic resistance challenges the need of an alternative treatment. Therefore, it is important to explore a solution to treat infection and the biofilm formed by *A. hydrophila.*

**Objectives:**

In this study, we used shallot skin powder and actinomycetes metabolite 20 PM as antimicrobe and antibiofilm to treated eels infected with *A. hydrophila*.

**Results:**

Shallot skin powder (6.25 g 100 g^−1^ feed) and Actinomycetes 20 PM metabolite (2 mL 100 g^−1^ feed) were found to be effective as antimicrobe and antibiofilm agent in treating eels infected with *A. hydrophila*. Eel treated with antibiotic, shallot skin powder, and actinomycetes metabolite had 80%, 66%, and 73% survival rates, respectively. Other indicators such as red blood cell count, hemoglobin, and hematocrit were increased, but white blood cell count and phagocytic activity were dropped. Biofilm destruction were analyzed using scanning electron microscopy to determined antibiofilm activity of actinomycetes metabolite against biofilm of *A. Hydrophila.*

**Conclusions:**

Shallot skin powder and actinomycetes metabolite were potential to treat infection of *A. hydrophila* in eel as an alternative treatment to antibiotics.

**Supplementary Information:**

The online version contains supplementary material available at 10.1186/s13104-023-06611-9.

## Introduction

Eel (*Anguilla bicolor bicolor*) contains high vitamin A [1] and vitamins B1, B2, B6, C, D, E, omega 3 [2], also Mg, Ca, Zn, and Fe [3]. Indonesia's eel production from 2019 to 2020 can meet around 25% of the world's eel demand [4, 5]. High-density cultivations are required to increase production, but it can pose a disease threat, including *A. hydrophila* infection which cause motile aeromonad septicemia disease with high mortality [6] and transmission rate [7, 8]. Treatment of antibiotics in aquaculture might leave residues in the environment, consumers, and products [9]. Natural compounds are required as an alternative solution. In this study we used shallot skin powder and actinomycetes metabolite. Shallot skin can inhibit pathogenic bacteria, due to pigments anthocyanins which belong to the class of flavonoid [10–12]. Anthocyanins act as antibacterial, antiviral, and antifungal and antioxidant activity [13, 14]. Metabolite of actinomycetes is derived from Actinomycetes isolates 20 PM. From our previous study, we found it showed antibiofilm activity against biofilm formed by *A. hydrophila*. The high polysaccharide content of actinomycetes extract inhibited and disrupted biofilm of *A. hydrophila* and reported no toxicity on aquatic organisms [15]. At a dose of 2 mL 100 g^−1^ of feed, the Actinomycetes supernatant is able to control biofilm for A. *hydrophila* that infect tilapia with a survival rate of 93.33% [16].

## Main text

### Methods

#### Fish and aquarium preparation

Eels of an average weight of 7.65 ± 0.32 g were obtained from Department of Aquaculture IPB University. We used 20 aquariums; five fishes were distributed into each aquarium with density of 212.5 g m^−2^.

#### Bacterial cultivation

We used *A. hydrophila* from infected eel from previous study. It was growth on brain heart infusion broth (Oxoid) and identified biochemically using Kit API 20NE (Biomeriux). The cultures were prepared in tryptic soy agar (Oxoid) with an overnight incubation at 28 °C. The concentration was adjusted to 10^8^ cells mL^−1^ for experimental use.

#### Feed preparation

The feed used was commercial feed FL 0, it was divided into 5 types according to the treatment, namely negative control (K−), positive control (K+), Enrofloxacin antibiotic (Enro), Shallot skin powder (KBM), and Actinomycetes metabolite 20 PM (Actino). The shallot skins were washed and dried without direct sunlight for 4 days, then processed to become powder. 6.25 g of shallot skin powder was added to 100 g of fish feed for KBM treatment. Two mL of Actinomycetes supernatant were added to 100 g fish feed for Actino treatment. Antibiotic control was prepared with 0.2 g of Enrofloxacin for 100 g of fish feed. We also prepared negative and positive control.

The feed was coated with the ingredients for each treatment. To agglutinate the feed and the treatment ingredients, 2% of tapioca flour was added. The modified feed was added with hot water and stirred until it became paste.

#### Challenge test and water quality measurement

The challenge test was carried out by intramuscular injection with 0.1 mL of *A. hydrophila* suspension (10^8^ cells mL^−1^). Next, the fish were kept and observed until the 14th day.

Measurement of temperature and pH of water was carried out every two days. While for dissolved oxygen and ammonia levels once a week. During maintenance, water temperature ranged from 26.0 to 27.8 °C, pH level from 7.01 to 7.69, DO levels from 4.35 to 5.5, while ammonia levels from 0.011 to 0.039 during rearing (Additional file 1: Table S1).

Blood and Immune Assays (Total Red Blood Cells Count, Total White Blood Cells Count, Hemoglobin, Phagocytic Activities and Respiratory Burst).

Blood draws were performed on days 0, 3, and 10 for all of blood assays. For total red blood cells count, calculation was done using haemocytometer and observed using microscopy [17]. Blood cells in 80 small boxes (5 large boxes) were counted.

$$\sum \mathrm{ Total \,Red \,Blood \,Cells \,Count }(\mathrm{sel \,mm}^{-3})= \frac{\sum \mathrm{ calculated \,cells }\times 4000 \times \mathrm{ diluent \,factor}}{\sum \mathrm{ small \,calculated \,boxes}}$$

For total white blood cells count calculation were done using haemocytometer and observed using microscopy. [17].

$$\sum \mathrm{ Total\, White \,Blood \,Cell \,Count }(\mathrm{sel \,mm}^{-3})=\frac{\sum \mathrm{ calculated \,cells}}{\sum \mathrm{ large \,calculated \,boxes }}\times 250\times \mathrm{diluent\, factor}$$

For hemoglobin, fish blood was taken using a Sahli pipette up to a line of 0.02, then inserted into a Sahli tube filled with HCl [17]. Hemoglobin levels were expressed in grams per 100 mL of blood (G%). For Hematocrit, we used hematocrit tube to be touched to the blood sample and filled until the tube up to ¾ part. The capillary pipe was centrifuged for 15 min, 3500×*g* [17].

$$\mathrm{Hematocrit }(\mathrm{\%})=\frac{\mathrm{long \,volume \,of \,red \,blood \,cells \,that \,settle}}{\mathrm{total \,length \,of \,blood \,volume \,in \,the\, tube}}\times 100\mathrm{\%}$$

While for phagocytic activity, A total of 50 μL of blood supplemented with 50 μL of *Staphylococcus aureus* 10^7^ CFU mL^−1^, then incubated at 28 °C for 20 min. Then 5 μL was taken to make a preparate review, fixed with 100% of methanol and dried, soaked in Giemsa's solution for 20 min, rinsed and dried. Observation was done using microscopy [18].

$$\mathrm{Phagocytic\, Activity }(\mathrm{\%})=\frac{\mathrm{total \,of \,cells \,that \,perform \,phagocytosis}}{\mathrm{total \,of \,phagocytic \,cells}}\times 100\mathrm{\%}$$

For respiratory burst, 50 μL of blood samples were inserted into a microplate well, incubated at 37 °C for 1 h. Then washed with 50 μL of PBS supplemented with 50 μL of 0.2% nitroblue tetrazolium reagent, incubated for 1 h. Then fixed using 50 μL of 100% methanol followed by 50 μL of 30% methanol, air-dried. 60 μL of potassium hydroxide and 70 μL of dimethylsulfoxide solution were added. Optical density observed using ELISA Reader 540 nm [19].

#### Microscopic observation of biofilm

Fish intestine samples were taken on day 14 for positive control, antibiotic, shallot skin powder, and metabolite of Actinomycetes treatments [20]. Scanning Electron Microscopy determination was done at National Research and Innovation Agency (BRIN).

### Results

#### Survival rate

From day 1 to day 14, the survival rate of eels was observed after being challenged with *A. hydrophila*. Eel survival decreased from day 1 to day 5 for positive control group, and treated group. During the rearing period, eel survival did not decrease in the negative control group (Additional file 1: Fig. S1).

**Fig. 1:**
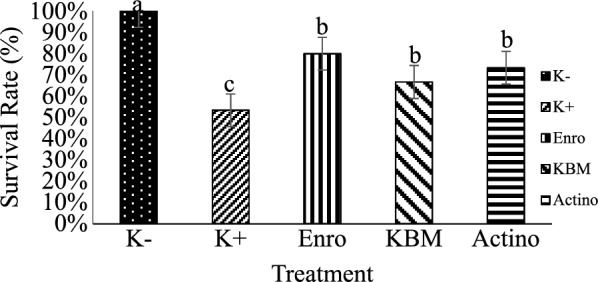
Survival rate of eel infected with *A. hydrophila* after being treated. K−: negative control; K+: positive control; Enro: Treatment with enrofloxacin; KBM: Treatment with shallot skin powder; Actino: Treatment with actinomycetes metabolite

The survival rate of eel showed significantly different (P < 0.05) in the negative control and treated group, but the value was not significantly different (P > 0.05) between each treated group (Fig. 1).

#### Blood and immune parameters

Total red blood cells count were observed from days 0, 3, and 10. During pre-challenge, it showed not significantly different (P > 0.05) between each group. While total red blood cells count value significantly different (P < 0.05) on day 3 between negative control and other group, but the KBM and Actino group were not significantly different (P > 0.05). Total red blood cells count on day 10 different (P < 0.05) between negative control and other group, but value between Enro and Actino group were not different (P > 0.05) (Additional file 1: Fig. S2).

Total white blood cells count observed from days 0, 3, and 10. During pre-challenge total white blood cells count of each group there is no different (P > 0.05). On day 3 the value were different (P < 0.05) between negative control and other group, but the value was not different (P > 0.05) between positive control and treated group. On day 10 showed different value (P < 0.05) between negative control and treated group, but the value of positive control compare with KBM group were not different (P > 0.05), The value of Enro compare with Actino group were not different (Additional file 1: Fig. S3).

The hemoglobin of eel was observed from days 0, 3, and 10. On day 3, it showed different (P < 0.05) between negative control and other treated group, but the value of KBM compare with Actino group were not different (P > 0.05). On day 10 it showed different value (P < 0.05) between negative control and other group, but the value of Enro compare with Actino group were not different (P > 0.05) (Additional file 1: Fig. S4).

Hematocrit were observed from days 0, 3, and 10. The hematocrit levels on day 3 showed different value (P < 0.05) between negative control and other group, but positive control was not different compare with treated group (P > 0.05). On day 10 performed different (P < 0.05) between negative control and other group, but the value between treated group were not different (P > 0.05) (Additional file 1: Fig. S5).

**Fig. 2:**
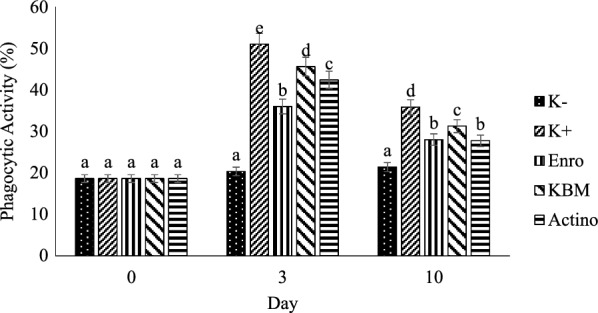
Phagocytic activity of eel during pre and post-challenge period of the *A. hydrophila* infection. K−: negative control; K+: positive control; Enro: Treatment with enrofloxacin; KBM: Treatment with shallot skin powder; Actino: Treatment with actinomycetes metabolite

The phagocytic activity of eel on day 3 showed different (P < 0.05) between negative control and other group. On day 10, it showed different (P < 0.05) between negative control and other group, but the value between Enro compare with Actino group were not different (P > 0.05) (Fig. 2).

Observations of respiratory burst of eel were done during pre and post challenge (day 0, 3, and 10). During pre-challenge, the value was not different (P > 0.05) between negative control and other group. While on day 3 different (P < 0.05) between negative control and other group, but it showed slightly different (P > 0.05) between each treated group. On day 10, it showed different (P < 0.05) between negative control compare with positive control and KBM group (Additional file 1: Fig. S6) [21].

#### Biofilm determination

**Fig. 3:**
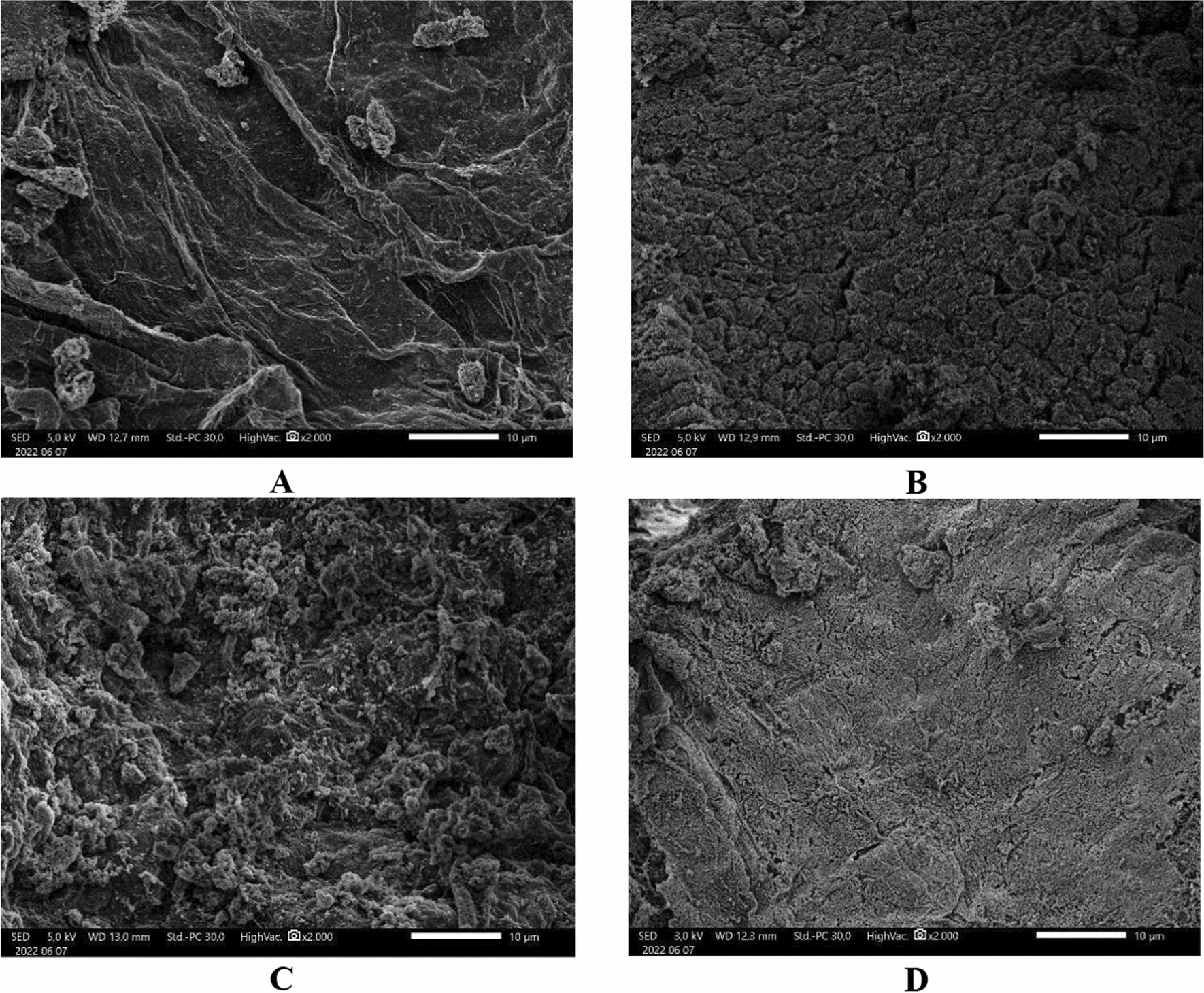
Scanning electron microscope observation of the intestine of eel after being challenge with *A. hydrophila* **A** positive control (K+), **B** treatment with enrofloxacin (Enro) **C** treatment with shallot skin powder (KBM), **D** treatment with actinomycetes supernatant (Actino)

Scanning Electron Microscopy determined that there was destruction of biofilm formation on actino group compare with positive control, while for enro group it performed destruction as well but not in treatment with KBM (Fig. 3).

## Discussion

The survival rate of eel after being challenge with *A. hydrophila* performed Enro, KBM, and Actino group were not different (P > 0.05). It might happen due to antibacterial activity from shallot skin which can inhibit the growth of pathogen [10]. Furthermore, the metabolite of actinomycetes 20PM capable to inhibit and destruct biofilm formation of *A. hydrophila* [22].

On day 3, total red blood cells count showed that positive control, Enro, KBM, and Actino group were decreasing. It might happen due to red blood cell lyses by *A. hydrophila* and disrupting the circulatory system [22]. While, on day 10, showed increased in Enro, KBM, and Actino group, due to immune system recovery phase [23].

On day 3, total white blood cells count was increased in positive control, Enro, KBM, and Actino groups. White blood cell play role as an active immune response against pathogenic bacteria [22]. On day 10, it showed lower in Enro, KBM, and Actino groups because infected eels go through an immune system recovery period [23].

Hemoglobin levels performed decreasing in positive control, Enro, KBM, and Actino group on day 3. Due to lyses of red blood cell by *A. hydrophila* reducing the oxygen level in red blood cell transported by hemoglobin [22]. While, on day 10, it increased in Enro, KBM, and Actino group, since eels go through an immune system recovery phase [24].

In the case of hematocrit level, we found decrease in positive control, Enro, KBM, and Actino group on day 3. Since decreasing of red blood cell level also affect hematocrit level [22]. On day 10, increasing in Enro, KBM, and Actino treatments. Since, eels infected with *A. hydrophila* go through an immune system recovery period [25].

Phagocytic activity of eel after being challenge revealed in positive control, Enro, KBM, and Actino group on day 3 were increased. *A. hydrophila* infection activate phagocytic cells as non-specific immune response [22]. On day 10, it was reduced in Enro, KBM, and Actino group. Eels infected with *A. hydrophila* go through immune system recovery period [26].

The respiratory burst revealed that positive control, Enro, KBM, and Actino were increased on day 3 but then reduced as a sign of recovery process. Phagocytic cells destroy the pathogens [27], level of oxygen in the phagocytic cell influences the phagocytic process of respiratory burst [15, 28]. SEM analysis revealed there is destruction of biofilm formation of *A. hydrophila* in actino group compare with positive control, since metabolite of actinomycetes 20PM have antibiofilm activity in vitro [15, 29]. In Enro group also showed less of biofilm formation, it might happen due to growth inhibition of *A. hydrophila* by this antibiotic. While for KBM we found there is no antibiofilm destruction, since shallot skin known as antimicrobe due to the flavonoid content [30, 31].

### Conclusion

Shallot skin powder and metabolite of actinomycetes 20 PM were effective in treating eel infected with *A. hydrophila* which showed from survival rates and blood test performance. Shallot skin powder have antimicrobe activity while metabolite of actinomycetes performed antibiofilm activity.

### Limitation

This research only tested eel infected with *A. hydrophila*, activities against other fish pathogenic bacteria need to be explored.

### Supplementary Information

**Additional file 1: Figure S1.** Daily survival rate of eel after being challenge with *A. hydrophila.* K−: negative control; K+: positive control; Enro: Treatment with enromycin; KBM: Treatment with shallot skin powder; Actino: Treatment with actinomycetes metabolite. **Figure S2.** Total erythrocyte of eel after being challenge with *A. hydrophila.* K−: negative control; K+: positive control; Enro: Treatment with enromycin; KBM: Treatment with shallot skin powder; Actino: Treatment with actinomycetes metabolite.**Figure S3.** Total leucocyte of eel after being challenge with *A. hydrophila.* K−: negative control; K+: positive control; Enro: Treatment with enromycin; KBM: Treatment with shallot skin powder; Actino: Treatment with actinomycetes metabolite.**Figure S4.** Hemoglobin of eel after being challenge with *A. hydrophila.* K−: negative control; K+: positive control; Enro: Treatment with enromycin; KBM: Treatment with shallot skin powder; Actino: Treatment with actinomycetes metabolite.**Figure S5.** Hematocrit of eel after being challenge with *A. hydrophila.* K−: negative control; K+: positive control; Enro: Treatment with enromycin; KBM: Treatment with shallot skin powder; Actino: Treatment with actinomycetes metabolite.**Figure S6.** Respiratory burst of eel after being challenge with *A. hydrophila.* K−: negative control; K+: positive control; Enro: Treatment with enromycin; KBM: Treatment with shallot skin powder; Actino: Treatment with actinomycetes metabolite.**Table S1.** Water quality maintenance parameters measured during challenge with *A*. *hydrophilia.*

## Data Availability

All data generated or analysed during this study are included in this published article [and its supplementary information files].
