# Supplementary material for: The potential of shallot skin powder and actinomycetes metabolites as antimicrobe and antibiofilm in the treatment of eel (Anguilla bicolor bicolor) infected with Aeromonas hydrophila
Source: BMC Res Notes. 2023 Nov 9;16:324. doi: 10.1186/s13104-023-06611-9 (PMC10634037; doi:10.1186/s13104-023-06611-9)
Supplement: Supplementary file 1 — Additional file 1: Figure S1. Daily survival rate of eel after being challenge with A. hydrophila. K−: negative control; K+: positive control; Enro: Treatment with enromycin; KBM: Treatment with shallot skin powder; Actino: Treatment with actinomycetes metabolite. Figure S2. Total erythrocyte of eel after being challenge with A. hydrophila. K−: negative control; K+: positive control; Enro: Treatment with enromycin; KBM: Treatment with shallot skin powder; Actino: Treatment with actinomycetes metabolite.Figure S3. Total leucocyte of eel after being challenge with A. hydrophila. K−: negative control; K+: positive control; Enro: Treatment with enromycin; KBM: Treatment with shallot skin powder; Actino: Treatment with actinomycetes metabolite.Figure S4. Hemoglobin of eel after being challenge with A. hydrophila. K−: negative control; K+: positive control; Enro: Treatment with enromycin; KBM: Treatment with shallot skin powder; Actino: Treatment with actinomycetes metabolite.Figure S5. Hematocrit of eel after being challenge with A. hydrophila. K−: negative control; K+: positive control; Enro: Treatment with enromycin; KBM: Treatment with shallot skin powder; Actino: Treatment with actinomycetes metabolite.Figure S6. Respiratory burst of eel after being challenge with A. hydrophila. K−: negative control; K+: positive control; Enro: Treatment with enromycin; KBM: Treatment with shallot skin powder; Actino: Treatment with actinomycetes metabolite.Table S1. Water quality maintenance parameters measured during challenge with A. hydrophilia. [file 13104_2023_6611_MOESM1_ESM.docx]

Figure S1 Daily survival rate of eel after being challenge with *A. hydrophila.* K-: negative control;K+:positive control; Enro: Treatment with enromycin; KBM: Treatment with shallot skin powder; Actino: Treatment with actinomycetes metabolite

Figure S2 Total erythrocyte of eel after being challenge with *A. hydrophila.* K-: negative control;K+:positive control; Enro: Treatment with enromycin; KBM: Treatment with shallot skin powder; Actino: Treatment with actinomycetes metabolite

Figure S3 Total leucocyte of eel after being challenge with *A. hydrophila.* K-: negative control;K+:positive control; Enro: Treatment with enromycin; KBM: Treatment with shallot skin powder; Actino: Treatment with actinomycetes metabolite

Figure S4 Hemoglobin of eel after being challenge with *A. hydrophila.* K-: negative control;K+:positive control; Enro: Treatment with enromycin; KBM: Treatment with shallot skin powder; Actino: Treatment with actinomycetes metabolite

Figure S5 Hematocrit of eel after being challenge with *A. hydrophila.* K-: negative control;K+:positive control; Enro: Treatment with enromycin; KBM: Treatment with shallot skin powder; Actino: Treatment with actinomycetes metabolite

Figure S6 Respiratory burst of eel after being challenge with *A. hydrophila.* K-: negative control;K+:positive control; Enro: Treatment with enromycin; KBM: Treatment with shallot skin powder; Actino: Treatment with actinomycetes metabolite

Table S1 Water quality maintenance parameters measured during challenge with *A*. *hydrophilia*

| Parameter | Treatment | | | | | Optimum | Reference |
| --- | --- | --- | --- | --- | --- | --- | --- |
|  | K- | K+ | Enro | KBM | Actino |  |  |
| Temperature (^o^C) | 26,6 - 27,0 | 26,2 - 27,3 | 26,0 - 27,2 | 26,0 - 27,8 | 26,5 - 27,0 | 23,0 - 31,0 | Scabra et al. 2016 |
| DO (mg/L) | 4,61 | 5,5 | 4,73 | 4,35 | 4,52 | > 4,0 | Scabra et al. 2016 |
| pH | 7,69 | 7,12 | 7,46 | 7,01 | 7,47 | 6,0 - 8,0 | Scabra et al. 2016 |
| Ammonia (mg/L) | 0,039 | 0,014 | 0,019 | 0,011 | 0,021 | < 0,1 | Scabra et al. 2016 |

**D**

**C**
